# Involvement of Degenerating 21.5 kDa Isoform of Myelin Basic Protein in the Pathogenesis of the Relapse in Murine Relapsing–Remitting Experimental Autoimmune Encephalomyelitis and MS Autopsied Brain

**DOI:** 10.3390/ijms24098160

**Published:** 2023-05-02

**Authors:** Chie Takano, Takuma Takano, Makoto Masumura, Ryuichi Nakamura, Shuichi Koda, Hiroki Bochimoto, Shigetaka Yoshida, Yoshio Bando

**Affiliations:** 1 Department of Functional Anatomy and Neuroscience, Asahikawa Medical University, Asahikawa 078-8510, Japan; 2 Department of Neurosurgery, Asahikawa Medical University, Asahikawa 078-8510, Japan; 3 Institute for Social Innovation and Cooperation, Niigata University, Niigata 951-8510, Japan; 4 Daiichi Sankyo Co., Ltd., Tokyo 140-8710, Japan; 5 Department of Cell Physiology, The Jikei University School of Medicine, Tokyo 105-8461, Japan; 6 Department of Anatomy, Akita University Graduate School of Medicine, Hondo 1-1-1, Akita 010-8543, Japan

**Keywords:** relapsing–remitting EAE, MBP, myelin morphology, relapse, ER stress

## Abstract

Multiple sclerosis (MS) is the chronic inflammatory demyelinating disease of the CNS. Relapsing–remitting MS (RRMS) is the most common type of MS. However, the mechanisms of relapse and remission in MS have not been fully understood. While SJL mice immunized with proteolipid protein (PLP) develop relapsing–remitting experimental autoimmune encephalomyelitis (RR-EAE), we have recently observed that some of these mice were resistant to the active induction of relapsing EAE after initial clinical and histological symptoms of EAE with a severity similar to the relapsing EAE mice. To clarify the mechanism of relapsing, we examined myelin morphology during PLP_139–151_-induced RR-EAE in the SJL mice. While RR-EAE mice showed an increased EAE severity (relapse) with CNS inflammation, demyelination with abnormal myelin morphology in the spinal cord, the resistant mice exhibited a milder EAE phenotype with diminished relapse. Compared with the RR-EAE mice, the resistant mice showed less CNS inflammation, demyelination, and abnormalities of the myelin structure. In addition, scanning electron microscopic (SEM) analysis with the osmium-maceration method displayed ultrastructural abnormalities of the myelin structure in the white matter of the RR-EAE spinal cord, but not in that of the resistant mice. While the intensity of myelin staining was reduced in the relapsing EAE spinal cord, immunohistochemistry and immunoblot analysis revealed that the 21.5 kDa isoform of degenerating myelin basic protein (MBP) was specifically induced in the relapsing EAE spinal cord. Taken together, the neuroinflammation-induced degenerating 21 kDa isoform of MBP sheds light on the development of abnormal myelin on the relapse of MS pathogenesis.

## 1. Introduction

Multiple Sclerosis (MS) is characterized as an autoimmune disease and causes the immune system to attack myelin and the nerves in the CNS, resulting in demyelination and axonal injury leading to serious neurologic damage in MS [1,2,3]. There are different types of MS including relapsing–remitting, primary progressive, and secondary progressive.

Relapsing–remitting MS (RR-MS) is the most common type of MS and is characterized by unpredictable relapses (attacks, flare-up, exacerbation) followed by partial or total remission (recovery) [4]. About 85% of people with MS are diagnosed with RRMS, which means that most MS patients will expect to experience relapses [5]. Previous studies have shown that there is a breach in the BBB during MS relapse, resulting in inflammatory demyelination and the making of new or larger lesions in the CNS [4]. However, the mechanisms of relapse and remission remain unknown. Therefore, it is required to identify the pathological features and molecular targets of the relapsing MS pathogenesis.

Experimental autoimmune encephalomyelitis (EAE) as an animal model for human MS is used to better understand the pathogenesis of MS and similar diseases such as Neuromyelitis Optica (NMO) and MOG IgG-associated disorder (MOGAD) with inflammatory demyelination in the CNS [6]. To address RR-MS pathogenesis, SJL/J mice immunized with the peptide of myelin proteolipid protein (PLP_139–151_) have often been used. In this model, PLP_139–151_-induced EAE (PLP-induced relapsing–remitting EAE; RR-EAE) symptoms are developed within 1–2 weeks, remitted thereafter, and then relapsed at 3–4 weeks after immunization, indicating that PLP-induced RR-EAE in SJL/J mice shares a certain characteristic with RR-MS [7]. The association of the reappearance of mononuclear cells was revealed in the relapse [8]. The expression of chemokines or cytokines is also observed in the relapse, indicating that further inflammatory attacks are implicated [9,10]. While RR-EAE is a useful model for the underlying pathology of MS, the detailed mechanisms causing relapse–remission in RR-EAE are not clearly understood. In the current study, we therefore attempted to understand the mechanisms of relapse–remission in RR-EAE.

Since we have found that some SJL/J mice immunized with PLP_139–151_ were resistant to developing EAE, we tried to understand what happened to the resistant mice in developing EAE. The current study was performed to identify the differences in myelin structures and glial responses between the relapsing and the resistant mice.

## 2. Results

### 2.1. Progression of the PLP-Induced RR-EAE

**Figure 1:**
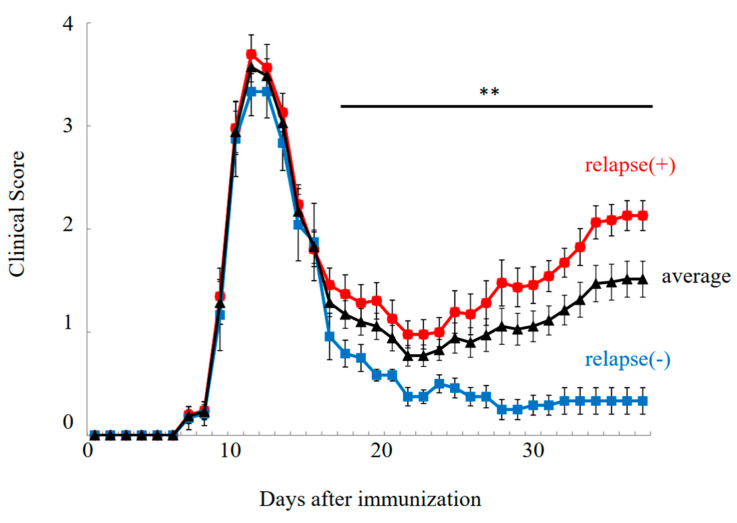
Disease course of RR-EAE. Progression of RR-EAE was daily monitored and scored on disease severity on a clinical scale from 0 to 5, as described in the text. EAE symptoms in relapse (−)/resistant mice (closed blue square) were significantly milder than that in relapse (+) mice (closed red circle). Average clinical score is shown in black line (closed triangle). Note all mice got severe EAE at the 1st episode. The mean clinical score (±SEM) is shown. ** *p* < 0.01 is shown (relapse (+) vs. relapse (−)).

PLP-immunized female SJL/J mice exhibited the relapsing–remitting EAE (RR-EAE) disease course (Figure 1, relapse (+)). In this model, EAE symptoms were developed on day 7 after immunization, and EAE scores reached a maximal mean grade on days 10–12. EAE symptoms were then remitted with lower EAE scores by day 20. However, EAE symptoms were relapsed around days 30–40 with a higher severity. While all mice experienced the first clinical episode (*n* = 36), twelve out of thirty-six female PLP-immunized SJL/J mice showed resistance to relapsing EAE, resulting in a milder EAE phenotype with diminished relapse (Figure 1, relapse (–)/resident).

### 2.2. Pathohistological Features in the PLP-Induced EAE Mice

**Figure 2:**
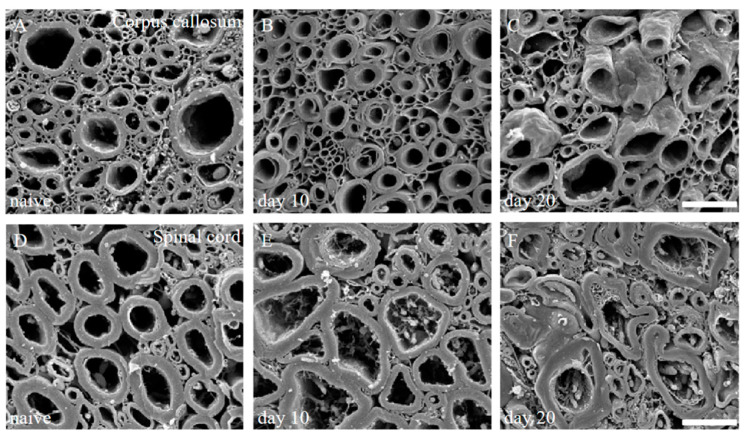
Myelin morphologies in PLP-induced EAE mice. Myelin morphologies in PLP-induced EAE mice were observed by SEM with osmium-maceration method. Corpus callosum (**A**–**C**) and spinal cord (**D**,**E**) were shown. While myelin morphologies in the corpus callosum of PLP-induced EAE (**A**–**C**) were relatively preserved, abnormal myelin morphologies were markedly observed in the spinal cord of PLP-induced EAE (**D**–**F**). Myelin detachment from the axon was detected from day 10 (**E**) to day 20 (**F**). However, these myelin abnormalities were not observed in 1st episode (**E**). Scar bars: 3 μm for (**A**–**C**), 6 μm for (**D**–**F**).

We have also previously reported that scanning electron microscope (SEM) analysis with the osmium-maceration method is a powerful tool for myelin research to assess demyelination including abnormal myelin morphologies, and that compact myelin detachment from the axon is observed at the initial phase of demyelination in EAE [11,12]. Both the brain (Figure 2A–C) and the spinal cord (Figure 2D–F) of the PLP-induced EAE mice were subjected to SEM analysis with the osmium-maceration method. Compared with the white matter of the spinal cord in naïve mice (Figure 2D), SEM analysis demonstrated that abnormal myelin morphologies such as myelin detachment from the axon and multiple myelin structures were observed in the white matter of the EAE spinal cord at the relapse phase (Figure 2F). Since these myelin abnormalities were not observed at day 10 (peak disease in first episode) (Figure 2E), these observations indicate that myelin abnormalities in the spinal cord are induced from the remission to the relapse. In contrast, myelin morphology in the corpus callosum of the PLP-induced EAE brain showed, at least partly, a typical morphology of demyelination in which myelin was gone (Figure 2B). However, the number of axons showing the typical demyelination were not as many, and the abnormal myelin morphologies seen in the RR-EAE spinal cord were not observed in the brain (Figure 2B,C). In additon, abnormal myelin morphologies likely to be in the EAE spinal cord were not seen in the brain (Figure 2B,C). In the present study, the RR-EAE pathogenesis in the spinal cord was then further investigated, rather than the brain.

**Figure 3:**
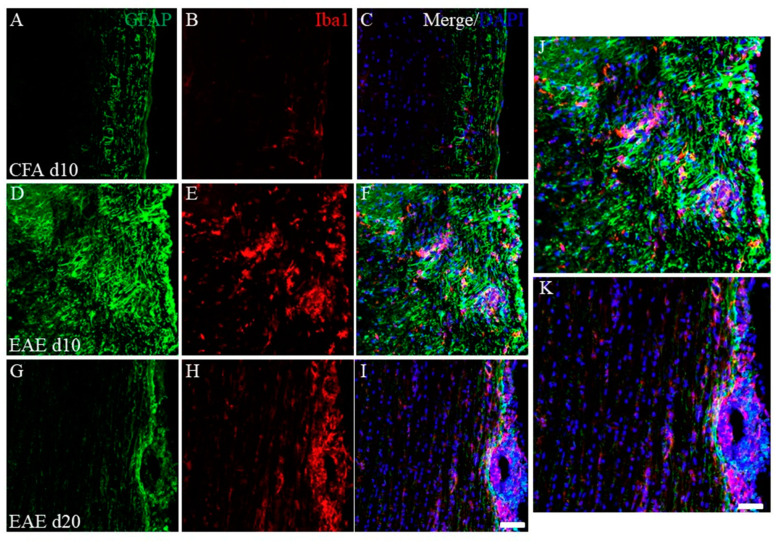
Increasing EAE-induced inflammatory cells at day 10 (peak disease). Frozen cross sections of spinal cords from CFA-injected control mice at day 10 post-immunization (1st episode, (**A**–**C**)) and PLP-induced EAE mice at day 10 (**D**–**F**,**J**) and day 20 after PLP-immunization (2nd episode, (**G**–**I**,**K**)) were stained with anti-GFAP (a marker for astrocytes, (**A**,**D**,**G**,**J**,**K**), green), anti-Iba1 (a marker for microglia/macrophage, (**B**,**E**,**H**,**J**,**K**), red) antibodies with DAPI ((**C**,**F**,**I**–**K**), blue). Merged images are shown (**C**,**F**,**I**–**K**). Images with a high-power magnification of panels (**F**,**I**) are shown (**J**,**K**). Scar bars: 500 μm.

To assess the EAE pathology, immunohistochemistry was performed next. As shown in Figure 3, immunohistochemistry revealed that the number of inflammatory foci reflected by DAPI staining was increased in the white matter of the EAE spinal cord at day 10 (peak disease) in the first episode (Figure 3F) after PLP-immunization, compared with the CFA-control (without PLP peptide, Figure 3C), indicating the increased number of infiltrating peripheral inflammatory cells into the spinal cord. On the other hand, most inflammatory cells reflected by DAPI staining demonstrated a restricted distribution at the pia matter of the EAE spinal cord at day 20 after PLP-immunization (remitting phase) (Figure 3I). Consistent with this observation, the accumulation of GFAP+ve astrocytes (Figure 3D,F,J) and Iba-1+ve microglia/macrophages (Figure 3E,F,J) were also found at the inflammatory sites of the white matter of the EAE spinal cord at day 10 after PLP-immunization, compared with the CFA-control (Figure 3A–C). On the other hand, the decreased number of GFAP+ve astrocytes and Iba-1+ve microglia/macrophages in the white matter of the EAE spinal cord was observed at day 20 after PLP-immunization (Figure 3G,H,K). In addition, the restricted distribution pattern of most Iba-1+ve peripheral macrophages was observed at the pia matter of the spinal cord at day 20 after PLP-immunization (Figure 3H,I,K).

### 2.3. Pathohistological Differences between RR-EAE and RR-EAE-Resistant Mice

**Figure 4:**
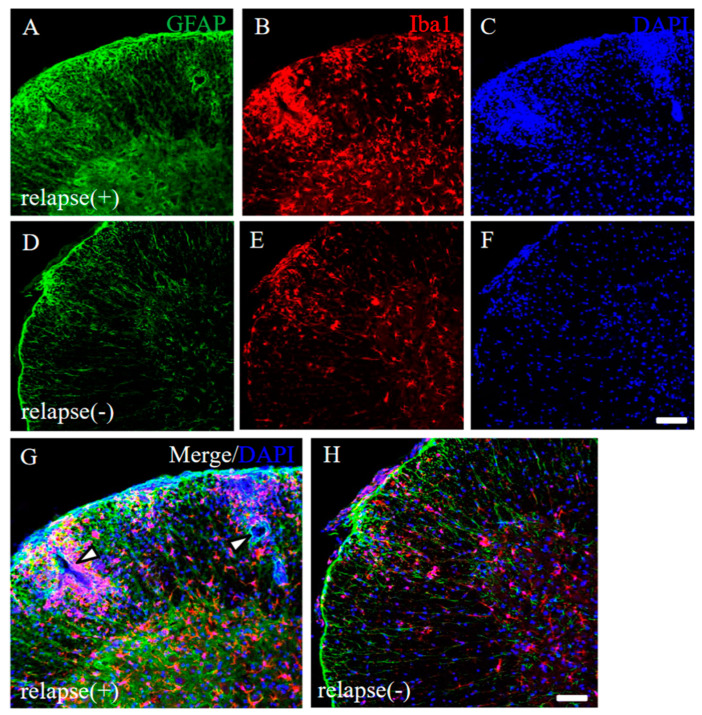
Pathological differences between RR-EAE and the resistant mice. Frozen cross sections of spinal cords from RR-EAE (relapse (+), (**A**–**C**)) and the resistant (relapse (−), (**D**–**F**)) mice at day 35–40 post-immunization were stained with anti-GFAP ((**A**,**D**), green) and anti-Iba1 ((**B**,**E**), red) antibodies. These sections were also stained with DAPI ((**C**,**F**), blue). Images with a high-power magnification of panels (**A**–**C**,**D**–**F**) are shown (**G**,**H**). (**G**) White arrowheads show severe inflammatory sites by recall infiltrating immune cells from the periphery. Scar bars: 500 μm.

To address the pathohistological differences between PLP-induced RR-EAE and RR-EAE-resistant mice, RR-EAE and the resistant mice were further investigated by immunohistochemistry, respectively. As we expected, the spinal cord in the RR-EAE mice showed severe inflammation by recall infiltrating immune cells from the periphery (Figure 4G, white arrowheads) and astrocytic and microglial gliosis with glial activation (relapse (+); Figure 4A–C,G). On the other hand, the resistant mice showed less inflammation in the CNS than that in the RR-EAE mice (relapse (−); Figure 4D–F,H). These results suggest that both peripheral infiltrating inflammatory cells and astrocytic and microglial gliosis were suppressed in the resistant mice.

### 2.4. EAE-Induced Increase of Angiogenesis in the RR-EAE Mice

**Figure 5:**
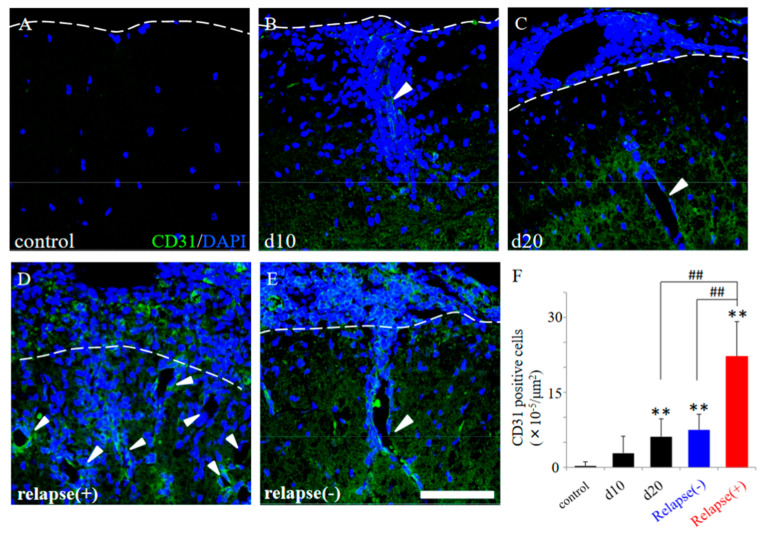
EAE-induced angiogenesis in the RR-EAE and the resistant mice. Frozen cross sections of spinal cords from the RR-EAE mice were stained with anti-CD31 antibody (a marker for angiogenesis, green). These sections were also stained with DAPI (blue). Each time course (control (**A**), day 10 (d10, (**B**)), day 20 (d20, (**C**)) are shown. Representative results from RR-EAE ((**D**), relapse (+)) and the resistant ((**E**), relapse (−)) are also shown. White arrowheads show a representative angiogenesis stained with anti-CD31 antibody. Panel (**F**) revealed that the number of CD31+ve cells in the RR-EAE, but not in the resistant mice, were significantly increased from remitting to relapse phase. ** *p* < 0.01 (vs. control), ## *p* < 0.01 (vs. relapse (+)) are shown. Scar bar: 50 μm.

**Figure 6:**
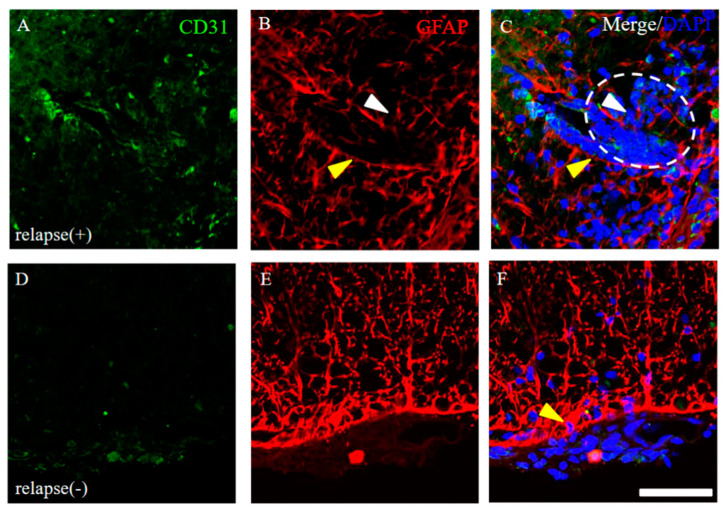
Distribution of GFAP+ve astrocytes around CD31+ve angiogenesis. Frozen cross sections of spinal cords from the RR-EAE mice were stained with anti-CD31 (green) and anti-GFAP (red) antibodies. These sections were also stained with DAPI (blue). Representative results from RR-EAE ((**A**–**C**), relapse (+)) and the resistant ((**D**–**F**), relapse (−)) were shown. Panels (**C**,**F**) are merged images. White arrowheads show infiltrating inflammatory cells from periphery through a gap in GFAP+ve astrocytic processes. White dashed circle shows an inflammatory site where peripheral immune cells reflected by DAPI staining are infiltrating into the CNS. Yellow arrowheads show tightly distributed GFAP+ve astrocytic processes. Scar bar: 50 μm.

Since the resistant mice had diminished infiltration of peripheral immune cells (partly relevant to showing as DAPI staining) into the CNS after PLP immunization (Figure 4), we next investigated the distribution pattern of CD31 expression in the EAE spinal cord. CD31, which are expressed in the endothelial cells of blood vessels, can be used specifically as a representative marker for angiogenesis [12,13,14,15]. As shown in Figure 5, many invasive peripheral inflammatory cells labeled with DAPI in the RR-EAE mice were mainly observed in the perivascular regions with CD31+ve angiogenesis (Figure 5B–E, arrowheads), compared with the control mice (Figure 5A). The increased number of CD31+ve cells, reflecting angiogenesis, were observed from day 20 after EAE induction and were further significantly increased from day 20 to the relapse phase (Figure 5F). Interestingly, the RR-EAE mice showed angiogenesis labeled with CD31 (Figure 5B–D, white arrowheads, Figure 5F and Figure 6A), whereas less angiogenesis was observed in the resistant mice (Figure 5E arrowhead, Figure 5F and Figure 6D). Since astrocytes are involved in the composition of the blood–brain barrier (BBB)/the blood–spinal cord barrier (BSCB), and a dysfunction of astrocytes leads to a breakdown of the BBB/BSCB and infiltration of inflammatory cells from the periphery, the distributions of GFAP+ve astrocyte expression were then examined in the RR-EAE and the resistant mice (Figure 6). Peripheral inflammatory cells from the RR-EAE mice infiltrated the white matter of the spinal cord through a gap in GFAP+ve astrocytic processes (Figure 6B,C, white arrowheads), but not the tight GFAP+ve astrocytic processes (Figure 6B,C, yellow arrowheads). In contrast, the distribution of the infiltrating peripheral inflammatory cells was restricted at the meninges in the resistant mice (Figure 6F, yellow arrowhead). Interestingly, GFAP+ve astrocytes in the resistant mice were tightly distributed at the meninges to dam up the infiltration of peripheral inflammatory cells into the white matter of the spinal cord (Figure 6E,F).

### 2.5. Distribution of Claudin-5 Is Sustained in the Resistant, but Not in the RR-EAE Mice

**Figure 7:**
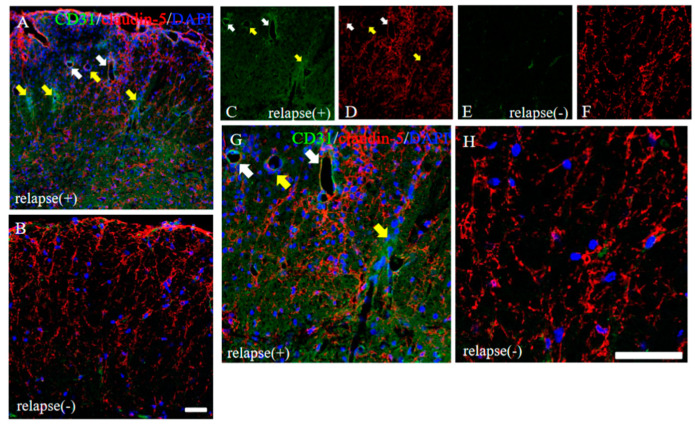
Distribution of claudin-5 is sustained in the resistant, but not in the RR-EAE, mice. Frozen cross sections of spinal cords from the RR-EAE (relapse (+)) and the resistant (relapse (−)) mice were stained with anti-CD31 ((**C**,**E**), green) and anti-claudin-5 ((**D**,**F**), red) antibodies. These sections were also stained with DAPI (blue). Merged images are shown (**A**,**B**,**G**,**H**). Images with a high-power magnification of panels (**A**,**B**) are shown (**G**,**H**). Yellow arrows show CD31+ve angiogenesis in areas with attenuated claudin-5 expression, indicating infiltrating sites of peripheral inflammatory cells. White arrows shows CD31+ve angiogenesis in area with sustained claudin-5 expression, indicating inhibiting inflammatory cells from the periphery. Scar bars: 50 μm.

To consider the possible machinery on BBB permeability in RR-EAE, the expression pattern of claudin-5 between the RR-EAE and the resistant mice was then investigated. As shown in Figure 7, the RR-EAE mice demonstrated that CD31+ve angiogenesis was observed in areas with attenuated claudin-5 expression, from which inflammatory cell infiltration was observed (Figure 7A,C,D,G, yellow arrows). On the other hand, inflammatory cell infiltration was blocked in areas where claudin-5 expression was maintained, even though CD31+ve angiogenesis was observed (Figure 7A,C,D,G, white arrows). In contrast, claudin-5 was tightly distributed in the spinal cord in the resistant mice (Figure 7B,F,H). These observations suggest that claudin-5-associated disruption of the BBB was induced from the remitting phase to the relapsing phase in the RR-EAE mice, but not in the resistant mice due to a sustained expression level of claudin-5.

### 2.6. Higher Expression of EAAT2, a Glutamate Transporter on Astrocytes, Was Observed in the RR-EAE Mice Rather than the Resistant Mice

**Figure 8:**
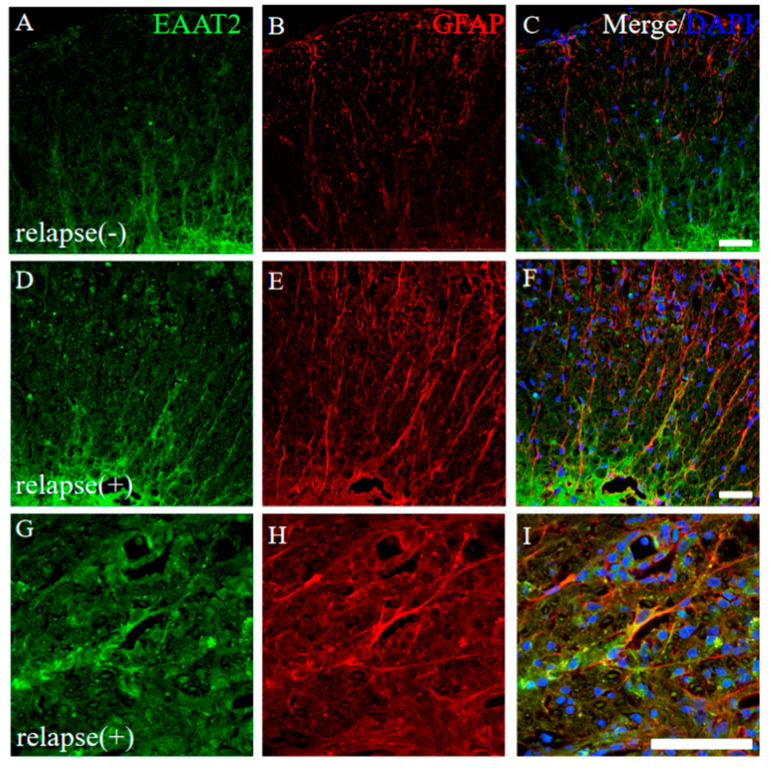
Higher expression of EAAT2 was observed in the RR-EAE mice rather than the resistant mice. Frozen cross sections of spinal cords from the RR-EAE (relapse (+)) (**D**–**I**) and the resistant (relapse (−)) mice (**A**–**C**) were stained with anti-EAAT2 (a glutamate transporter on astrocytes; (**A**,**D**,**G**), green) and anti-GFAP ((**B**,**E**,**H**), red) antibodies. These sections were also stained with DAPI (blue). Merged images are shown (**C**,**F**,**I**). Images with a high-power magnification of panels (**D**–**F**) are shown (**G**–**I**). Scar bars: 50 μm.

Glutamate toxicity is the major cause of the pathogenesis of EAE [16]. The expression of EAAT2, a glutamate transporter on astrocytes, was further examined. In the resistant mice, a lower expression of EAAT2 on GFAP+ve astrocytes was observed in the white matter of the EAE spinal cord (Figure 8A–C). In contrast, the increased expression level of EAAT2 on GFAP+ve astrocytes was observed in the white matter of the RR-EAE mice (Figure 8D–I). In particular, higher expression of EAAT2 on astrocytes was detected at the inflammatory sites where the accumulation of the inflammatory cells reflecting DAPI staining was found (Figure 8G–I). These results indicate that a higher expression of astrocytic EAAT2 was induced to remove glutamate toxicity in the RR-EAE spinal cord.

### 2.7. Severe Demyelination and Axonal Degeneration Were Observed in the RR-EAE, but Not in the Resistant Mice

**Figure 9:**
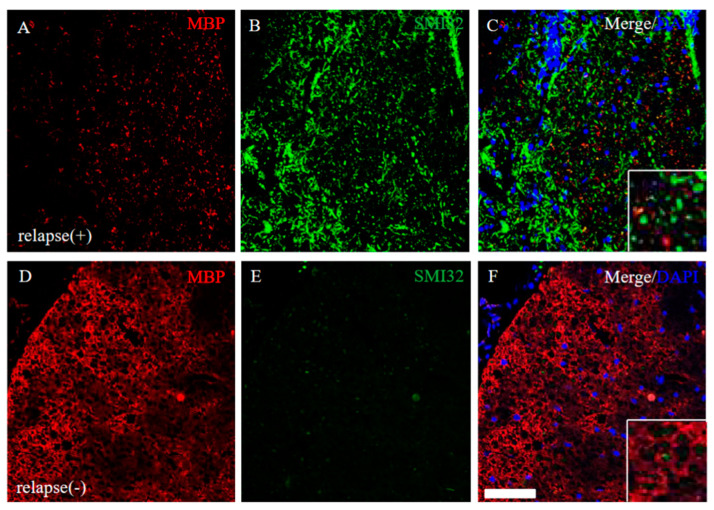
Severe demyelination and axonal degeneration were observed in the RR-EAE, but not in the resistant mice. Frozen cross sections of spinal cords from the RR-EAE (relapse (+)) (**A**–**C**) and the resistant (relapse (−)) mice (**D**–**F**) were stained with anti-MBP ((**A**,**D**), red) and anti-SMI32 ((**B**,**E**), green) antibodies. These sections were also stained with DAPI (blue). Merged images are shown (**C**,**F**). Images with a high-power magnification of panels are shown in the inset, respectively. Scar bar: 50 μm.

EAE-induced demyelination and axonal degeneration in the relapse phase were next explored. The RR-EAE mice revealed that down-regulation of MBP expression and up-regulation of SMI32 (a marker for axonal degeneration) was observed (Figure 9A–C), whereas MBP expression was not markedly changed and SMI32 immunoreactivity was rare in the RR-EAE resistant mice (Figure 9D–F). These results suggest demyelination and axonal degeneration by the relapsing EAE.

### 2.8. Immunoreactivity for Oligodendroglial and Their Progenitor’s Marker Protein was Changed in RR-EAE Mice

**Figure 10:**
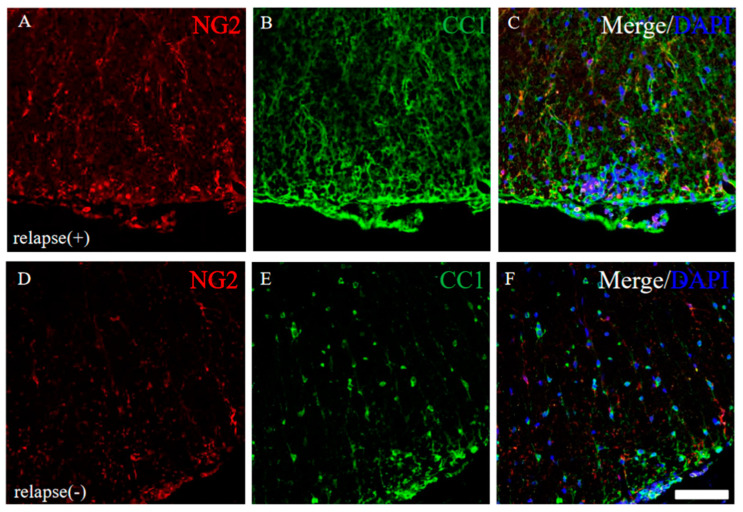
Immunoreactivity for oligodendroglial and their progenitor was changed in RR-EAE mice. Frozen cross sections of spinal cords from the RR-EAE (relapse (+)) (**A**–**C**) and the resistant (relapse (−)) mice (**D**–**F**) were stained with anti-NG2 ((**A**,**D**), red) and anti-CC1 ((**B**,**E**), green) antibodies. These sections were also stained with DAPI (blue). Merged images are shown (**C**,**F**). Scar bar: 50 μm.

Since it has been considered that EAE-induced demyelination is accomplished by oligodendroglial cell loss [17,18], the expression of CC-1 (a marker for oligodendrocytes) and NG2 (a marker for oligodendrocyte progenitors) were examined in the RR-EAE and the resistant mice. Immunohistochemistry revealed that the intensity of CC-1 and NG2 immunoreactivity was higher in the RR-EAE mice (Figure 10A–C) rather than the resistant mice (Figure 10D–F), even though the expression level of MBP was decreased in the RR-EAE mice (Figure 9A). However, the distribution patterns of CC-1 and NG2 expression in the spinal cord in the RR-EAE mice were different from that in the resistant mice. These observations provide a considerable hypothesis that immunoreactivity and the sensitivity of oligodendroglial and their progenitor’s marker proteins, such as CC-1 and NG2, are changed by the dysfunction of oligodendroglial lineage cells or uncertain reasons in the RR-EAE.

### 2.9. Induced the 21.5 kDa Isoform of MBP during Relapsing Phase was Degenerated in RR-EAE

**Figure 11:**
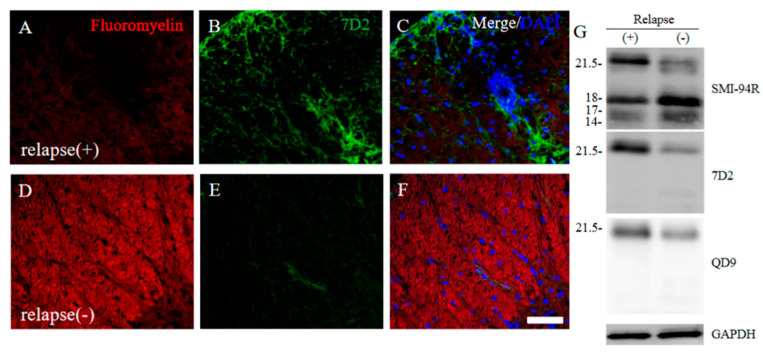
21.5 kDa isoform of MBP during relapsing phase was degenerated in RR-EAE. RR-EAE and resistant mice were perfused and immunostain was performed with Frozen cross sections of spinal cords from the RR-EAE (relapse (+)) (**A**–**C**) and the resistant (relapse (−)) mice (**D**–**F**) were stained with Fluoromyelin ((**A**,**D**), red) and anti-7D2 (a marker for 21.5 kDa isoform of MBP; (**B**,**E**), green) antibody. These sections were also stained with DAPI (blue). Merged images are shown (**C**,**F**). Scar bar: 50 μm. (**G**) Immunoblot analysis stained with anti-SMI-94R (a marker for MBP), anti-7D2 antibody, anti-QD9 antibody (a marker for degenerating MBP), and anti-GAPDH antibody (as an internal control). Note that anti-7D2 antibody specifically recognizes only 21.5 kDa isoform of MBP.

**Figure 12:**
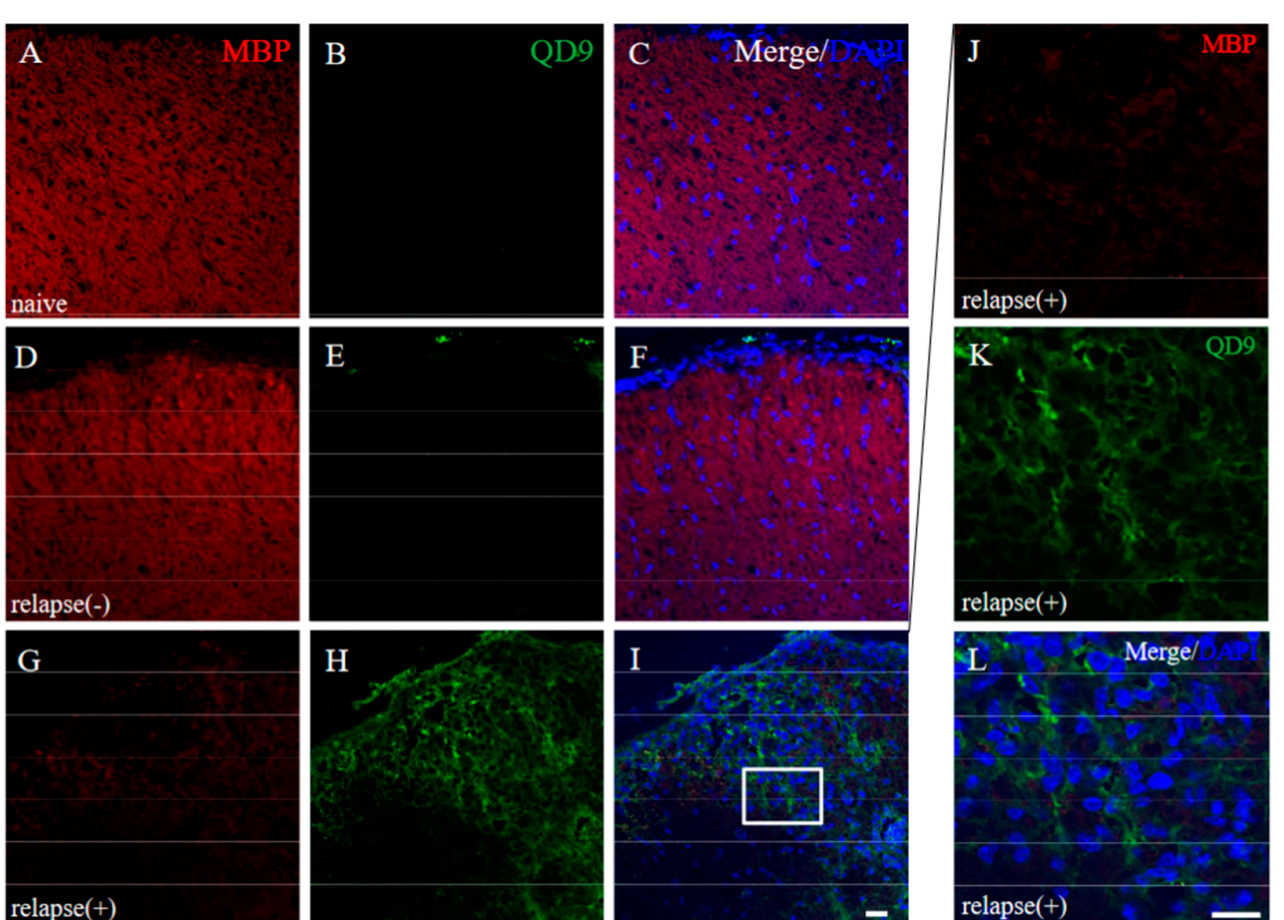
Immunoreactivity of degenerating MBP was increased in RR-EAE. Frozen cross sections of spinal cords from naïve (**A**–**C**), the resistant (relapse (−)) (**D**–**F**), and the RR-EAE (relapse (+)) mice (**G**–**L**) were stained with anti-MBP ((**A**,**D**,**G**,**J**), red) and anti-QD9 ((**B**,**E**,**H**,**K**), green) antibodies. These sections were also stained with DAPI (blue). Merged images are shown (**C**,**F**,**I**,**L**). Images with a high-power magnification of panels ((**I**), white square) are shown (**J**–**L**), respectively. Scar bar: 50 μm.

**Figure 13:**
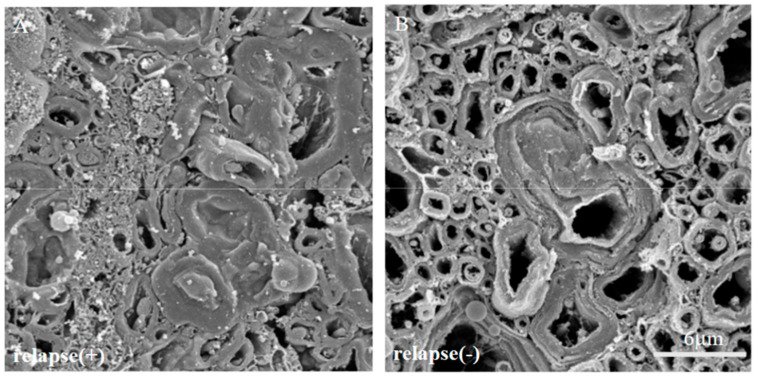
The ultrastructural myelin abnormalities and axonal degeneration were frequently observed in the relapsing EAE. The SEM analysis with osmium-maceration method showed differences ultrastructural myelin abnormalities and axonal degeneration between relapsing EAE mice (**A**) and the resistant mice (**B**).

Since immunoreactivity of CC-1 was changed in the RR-EAE mice (Figure 10), the expression of MBP was next examined a little more closely in the RR-EAE and the resistant mice. Similar to the result in Figure 9, fluoromyelin staining used as myelin staining likely to Luxol fast blue staining showed EAE-induced demyelination in the RR-EAE mice (Figure 11A), but not in the resistant mice (Figure 11D). In contrast, surprisingly, the immunoreactivity of the 21.5 kDa isoform of MBP stained with the anti-7D2 antibody, which can specifically recognize only the 21.5 kDa isoform of MBP, was up-regulated in the RR-EAE mice (Figure 11B), but not in the resistant mice (Figure 11E). Furthermore, immunoblot analysis clearly showed that the expression level of the 21.5 kDa isoform of MBP was increased in the RR-EAE mice, but not in the resistant mice (Figure 11G). In addition, a similar result was obtained by immunoblot analysis stained with the anti-7D2 antibody (Figure 11G). Moreover, immunoblot analysis using an anti-QD9 antibody, a marker for degenerating MBP [19,20], also showed the RR-EAE mice had a higher level of degenerating the 21.5 kDa isoform of MBP, compared with the resistant mice (Figure 11G). Besides this result, immunohistochemistry exhibited that immunoreactivity of the anti-QD9 antibody in the RR-EAE mice (Figure 12G–L), but not in the naive (Figure 12A–C), and the resistant mice (Figure 12D–F), and was detected in the spinal cord where MBP immunostaining was decreased. Further, the SEM analysis also supported our current results that the ultrastructural myelin abnormalities and axonal degeneration were frequently observed in the relapsing EAE mice (Figure 13A), whereas few abnormal myelin structures were observed in the resistant mice (Figure 13B).

### 2.10. 21.5 kDa Isoform of MBP Was Detected in the Demyelinating Lesion of Autopsied Human MS Patient’s Brain

**Figure 14:**
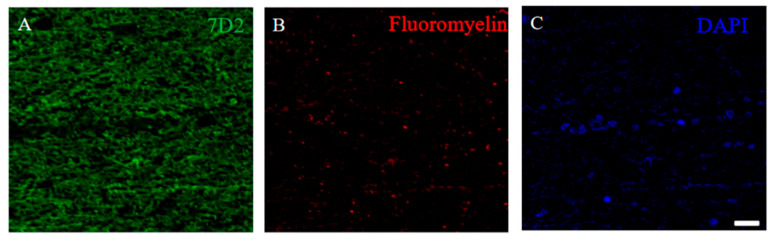
21.5 kDa isoform of MBP was detected in the demyelinating lesion of autopsied human MS patient’s brain. Immunostaining was performed on the autopsied human MS brain with anti-7D2 antibody ((**A**), green), Fluoromyelin ((**B**), red), and DAPI ((**C**), blue).

To assess the implication of the 21.5 kDa isoform of MBP in the pathogenesis of the RRMS, the autopsied human MS brain was further investigated. Immunohistochemistry showed the immunoreactivity of the anti-7D2 antibody in the demyelinating lesion where FM staining was decreased (Figure 14).

### 2.11. ER Stress Implicated the Induction of 21.5 kDa Isoform of MBP in Cultured Oligodendroglial Cells

**Figure 15:**
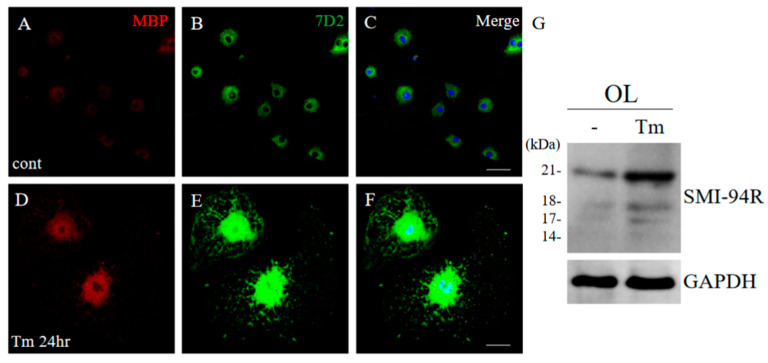
ER stress implicated the induction of 21.5 kDa isoform of MBP. Cultured oligodendrocytes (OLs) treated with/without Tm as an ER stress inducer for 24 h were performed immunocytochemistry with anti-MBP antibody ((**A**,**D**), red), and anti-7D2 antibody ((**B**,**E**), green). Merged images with DAPI (blue) were shown (**C**,**F**). Scar bar: 50 μm. Immunoblot analysis stained with anti-SMI-94R and anti-GAPDH antibody (**G**) (as an internal control).

To examine the inducible mechanism of the 21.5 kDa isoform of MBP, the ES cell-derived oligodendroglial progenitor cells (OPCs) were differentiated into oligodendrocytes (OLs) with a thyroid hormone (T3) incubation [12]. At day 7 after incubation with T3, OLs were treated with/without tunicamycin (Tm) as an ER stress inducer for 24 h. Immunocytochemistry demonstrated that Tm induced the expression level of MBP in OLs, compared with the control (Figure 15A,D). In addition, Tm-treated OLs showed morphological changes such as enlargement (Figure 15A,D). Moreover, the expression level of 21.5 kDa isoform of MBP was also increased in Tm-treated OLs. These results indicate that ER stress is involved in the formation of abnormal myelin structures mediated with 21.5 kDa isoform of MBP (Figure 15E,G).

## 3. Discussion

This study revealed that pathological changes, such as infiltration of peripheral inflammatory cells, astrocytic and microglial gliosis, angiogenesis, and morphological myelin abnormalities, occurred during remission to relapse. The current study also proposes a new possible hypothesis that degenerating 21.5 kDa MBP is involved in the mechanism of relapse in MS. In addition, ER stress was found to be implicated in the induction of degenerating 21.5 kDa MBP. According to a series of our results, this study provides a new insight into ER stress-mediated 21.5 kDa MBP playing a critical role in the pathogenesis of RR-EAE. As far as the authors know, this is the first report to investigate the detailed pathohistological changes during remission to relapse in the murine RR-MS model.

A previous study has revealed that one of the pathological features in PLP-induced RR-EAE is characterized by demyelination with the infiltrating peripheral inflammatory cells including encephalitogenic T cells into the CNS [21]. However, the mechanism by which recurrence occurs has remained unclear.

Our RR-EAE model exhibited that one-third of the PLP-immunized mice did not relapse (Figure 1). This result was informative, indicating that this model could be a useful tool to approach the elucidation of relapse mechanisms by comparing between the relapsing EAE mice and the resistant mice.

Ultrastructural SEM analysis demonstrated that pathological changes were more severe in the spinal cord rather than in the brain (Figure 2). The present study then focused on pathohistological changes in the spinal cord. As expected, the infiltrating peripheral inflammatory cells and astroglial and microglial activation referred to their gliosis were observed in the spinal cord in the first episode of PLP-EAE (Figure 3D–F,J). In contrast, there was an accumulation of inflammatory cells in the meninges, but no such pathohistological changes were observed in the white matter of the spinal cord during the remission phase (Figure 3G–I,K). Interestingly, the severe infiltration of peripheral inflammatory cells into the spinal cord and gliosis were recalled in the relapsed mice, whereas these inductions were suppressed in the resistant mice (Figure 4). As shown in Figure 5, the infiltrating inflammatory cells into the CNS, as reflected by DAPI staining, significantly increased from remission to relapse. Thus, a greater infiltration of peripheral inflammatory cells was suggested to involve a disruption of the BBB (perhaps, we should call it the blood–spinal barrier, but we will here refer to it as the BBB) or angiogenesis in the white matter of the spinal cord.

The molecular mechanisms related to angiogenesis and the BBB breakdown followed by the peripheral inflammatory cell infiltration were further investigated. As shown in Figure 5, the increasing numbers of angiogenesis indicated by immunostaining with the anti-CD31 antibody were observed in the relapse mice, but not in the resistant mice. In addition, an accumulation of peripheral inflammatory cells was observed in the sites where the perivascular astrocyte end feet were not stained with the anti-GFAP antibody around CD31+ve endothelial cells (Figure 6B,C, white arrowhead). In contrast, no inflammatory cell infiltration was observed in the sites where the perivascular astrocyte endo feet were tightly stained with the anti-GFAP antibody in the relapse mice (Figure 6E,F, yellow arrowhead). In the resistant mice, peripheral inflammatory cells remained on the meninges by the presence of astrocyte in the tight (Figure 6E,F).

Further, our results also clarified that claudin-5 plays a critical role in the mechanism of relapse. Claudin-5 regulates BBB permeability because claudin-5 is observed to be a key component of the tight junction and determines the sealing properties of the BBB [22]. The expression of claudin-5, and changes in its distribution, were then examined in the relapsed and the resistant mice. Similar to the results in Figure 6, infiltrating inflammatory cells from the periphery were observed in areas where there was no claudin-5 immunoreactivity, and no inflammatory cell infiltration was observed in areas where claudin-5 was detected (Figure 7). These observations suggest that the reduced expression level of claudin-5 could not maintain the BBB structure and triggered the infiltrating peripheral inflammatory cells into the CNS. Besides these findings, the relapsed mice had a higher expression of EAAT2 on astrocytes at the inflammatory sites (Figure 8G–I). Since EAAT2 is an astrocytic glutamate transporter to remove extracellular glutamate, the higher expression of EAAT2 in the relapsed mice indicates higher glutamate toxicity in the RR-EAE spinal cord. In contrast, the lower expression of EAAT2 in the resistant mice indicates lower glutamate toxicity in the spinal cord.

The current study also provides a new mechanism of demyelination during the relapsing EAE. As shown in Figure 9, demyelination and axonal degeneration was observed in the relapsed mice, but not in the resistant mice. This result was supported by the above results and could be easily expected. Nevertheless, surprisingly, the expression levels of oligodendrocyte lineage markers NG2 and CC1 increased rather than decreased, and their expression patterns were obviously altered in the relapsed mice, but not in the resistant mice (Figure 10). As far as the authors know, this is the first report to evidence their altered staining patterns. Several lines of evidence have suggested that oligodendrocyte death induced by inflammatory attacks contributes significantly to the development of MS and EAE [23,24,25]. Oligodendroglial apoptosis has been identified as the earliest structural change in newly forming demyelinating lesions in both MS and EAE by a number of studies [23,24,25]. Although the cause is obscure, the abnormal myelin structures during demyelination are not stained by LFB staining or other methods. Therefore, it is generally interpreted that the myelin sheath is gone due to oligodendroglial cell death. However, our previous ultrastructural studies have clearly shown that typical demyelination by cuprizone eliminates oligodendrocytes, but EAE-induced demyelination does not necessarily eliminate oligodendrocytes, and abnormal myelin structures are more frequent than oligodendroglial cell death [11,12,26]. Therefore, we have proposed a revised concept that demyelination includes abnormal myelin structures [11]. Since it is speculated that staining sensitivity may have been changed by the morphological abnormalities/dysfunctions of oligodendrocytes and their progenitor cells, myelin morphology was further investigated. As expected in general, the relapsed mice exhibited decreased fluoromyelin staining indicating demyelination in the relapsed mice, while the resistant mice showed sustained staining with fluoromyelin (Figure 11). However, most amazingly, immunohistochemistry with the anti-7D2 antibody, that detects only 21.5 kDa isoform of MBP, showed obvious staining only in the spinal cord, where fluoromyelin staining was decreased in the relapsed mice (Figure 11). This result is also supported by immunoblot analysis (Figure 11G). Moreover, immunoblot analysis with the anti-QD9 antibody, that detects demyelinating MBP, demonstrated that the induced 21.5 kDa isoform of MBP had degenerated in the relapsed mice (Figure 11G). This result is also supported by immunohistochemistry with the anti-QD9 antibody (Figure 12) and ultrastructural SEM analysis (Figure 13). Immunoreactivity stained with the anti-QD9 antibody was also detected in the autopsied RRMS patient’s brain (Figure 14). Again, as far as the authors know, this is the first report of direct evidence on the presence of degenerating MBP during the relapse phase. Further study will be required for understanding the detailed mechanism that induces these pathological changes.

Finally, the current study proposed a hypothesis that ER stress mediated pathogenesis regarding degenerating MBP during the relapse phase. The activation of the UPR followed by ER stress has been observed in multiple cell types in MS and EAE lesions [23,27,28,29]. We have previously reported that ER stress plays a pivotal role in the pathogenesis of various neurodegenerative diseases [30,31,32,33,34,35,36,37]. While there is no denying that inflammation is the ultimate cause of neurodegeneration in MS and EAE, it is also true that the process leading from an immune attack on oligodendrocytes and myelin to neurodegeneration remains largely elusive. In fact, we have previously reported that anti-MOG autoantibodies elicit ER-stress against oligodendrocytes in the murine MOG-induced EAE model. Our findings suggest that ER-stress is involved in the pathogenesis of multiple sclerosis. The current study then shed light on the effects of ER stress on pathological changes in oligodendrocytes. Namely, this study focused on the expression patterns of MBP, one of the myelin sheath component proteins, by ER-stress. As shown in Figure 15, Tm induced the 21.5 kDa isoform of MBP expression with a dramatic enlargement of oligodendroglial morphological changes. Unlike the cytoplasmic pattern of MBP stained with an anti-MBP antibody in the normal condition (Figure 15D), interestingly, immunoreactivity staining with an anti-7D2 antibody in Tm-treated cultured oligodendrocytes was mainly observed in the nucleus rather than the cytoplasm (Figure 15E). This result suggests that, at least partly, ER stress implicates the induction of the 21.5 kDa isoform of MBP and morphological changes of oligodendrocytes. By extension, ER stress may also be involved in the formation of the abnormal myelin structures which are observed in the relapsed mice. Our previous reports have demonstrated the implications of well-developed ER and the accumulation of mitochondrion in the pathogenesis of EAE [11,26]. In addition, we have previously clarified that the anti-myelin oligodendrocyte glycoprotein (MOG) autoantibody can also cause ER stress to drive oligodendroglial enlargement in cultured oligodendrocytes [12]. In addition, the relapsed mice had an increased expression level of the 21.5 kDa isoform of MBP (Figure 11G). In contrast, the resistant mice showed the enriched expression of the 18.5 kDa isoform of MBP and the lower expression of the 21.5 kDa isoform of MBP (Figure 11G). MBP has four isoforms ranging in normal molecular mass from 14 kDa to 21.5 kDa [38]. Although the function of the 21.5 kDa MBP isoform has still not been elucidated and has been enigmatical, the altered expression of 21.5 kDa MBP in demyelinating pathologies has been reported [39]. As previously reported [38], Harauz and Boggs have reviewed that the 21.5 kDa MBP isoform is the first of the classic isoforms to be synthesized, starting with oligodendrocyte progenitor cells. It is nuclear-targeted and serves to increase oligodendrocyte proliferation and induce the secretion of soluble factors (potentially NGF and/or BDNF) to enhance neurite outgrowth. It is also up-regulated in remyelination attempts in multiple sclerosis [38], like Golli [40,41], so presumably is involved in early events of oligodendrocyte differentiation and/or proliferation. The 18.5 kDa MBP isoform comes next, being synthesized in copious amounts as the membrane processes are extended, and ensheathing the axon. Myelin basic protein is observed only in oligodendrocytes that have migrated into axonal pathways, and it is produced just before the commencement of axonal ensheathment [40,41]. In mature myelinating oligodendrocytes, the 18.5 kDa MBP has been considered to redistribute from the soma and primary processes into the myelin sheaths, reflecting a change in the site of MBP mRNA expression [42,43]. Therefore, the 18.5 kDa isoform, which predominates in adult CNS myelin is generally considered to be essential for its development and stability [38,44,45,46,47,48]. Considering our data and previous reports, the 21.5 kDa isoform of MBP introduced by the relapse phase in RR-EAE and tunicamycin in the cultured oligodendrocytes may be induced to attempt the remyelination process. However, it is a possible hypothesis that ER stress degenerates the induced 21.5 kDa isoform of MBP and enhances abnormal myelin structures resulting in the suppression of remyelination. These results suggest that the control of ER stress-induced relapse may be the key to preventing relapse. Future investigations of the temporal and spatial distribution and function of MBP isoforms in the relapsing–remitting EAE/MS will be pivotal in enhancing our understanding of essentially promoting remyelination in MS and preventing MS relapse.

## 4. Material and Methods

### 4.1. Animals

Female SJL/J mice (purchased from Charles River, Yokohama, Japan) were used at 6–8 weeks of age [49]. The experimental procedure was approved by either the Institutional Committee for Experimental Animals (Asahikawa Medical University, Asahikawa and Asubio Pharma Co., Ltd., Kobe, Japan, respectively. #14152, 15194, 16179, 16180).

### 4.2. EAE Induction

Proteolipid protein peptide (PLP_139–151_; HSLGKWLGHPDKF, scrum, Tokyo, Japan)-induced RR-EAE (RR-EAE) was performed based on previously described [49]. In brief, SJL/J mice were immunized s.c. in the flank with an emulsion made of 75 μL of Ag peptide (50 μg of PLP_139–151_) and 75 μL of complete Freund’s adjuvant containing 0.4 mg of heat-inactivated *Mycobacterium tuberculosis* (H37Ra; Difco Laboratories, Franklin Lakes, NJ, USA). Each animal also received 200 ng of pertussis toxin (Sigma Aldrich, St. Louis, MO, USA) through i.p. injection on days 0 and 2 post-immunization. EAE clinical score was determined in a blinded fashion as described previously [11,12]: 0, no paralysis; 0.5, stiff tail; 1, limp tail or isolated weakness of gait without a limp tail; 2, partial hind limb paralysis; 3, total hind limb or partial hind and front limb paralysis; 4, total hind leg and partial front leg paralysis; and 5, moribund or dead animal. A mean clinical score was assigned to each group using this scale and used for statistical analysis (Mann–Whitney U-test).

### 4.3. Immunohistochemistry

Animals were sacrificed and perfused with cold PBS followed by 4% paraformaldehyde (PFA) in 0.1 M phosphate buffer (PB, pH 7.4) [11,12]. Either brain or spinal cord was then removed, immersed in 30% sucrose in 0.1 M PB for 1–2 days, and frozen in OCT medium. Frozen 14 μm sections were prepared on a cryostat, and stored at −30 °C until use. Human autopsied brain tissues in MS patients were obtained from Tissue Brain Bank in Maryland University (College Park, MD, USA) [12]. Human autopsied brain tissue blocks were embedded on paraffin in a standard protocol. The sections were cut by a microtome, followed by histological studies. For immunohistochemistry with paraffin sections, antigen retrieval was also performed by a specific reagent (Immunosaver, FUJIFIRM WAKO, Osaka, Japan) [12]. Fluorescence staining for myelin was done using FluoroMyelin Red (FM; a lipophilic stain for compact myelin, Molecular Probes/Invitrogen, Eugene, OR, USA) following a manufacturer’s protocol [50]. For Immunohistochemistry, the sections were immunostained with either anti-MBP antibody (1:1000, SMI-94R, Biolegend, San Diego, CA, USA), anti-MBP antibody (1:1000, MCA-7D2, Encor, Gainesville, FL, USA), anti-phospho-MBP antibody (1:1000, Merck Millipore, Darmstadt, Germany), anti-degenerating MBP antibody (1:1000, QD9, Merck Millipore, Darmstadt, Germany), anti-SMI32 antibody (1:1000, Biolegend, San Diego, CA, USA), anti-NG2 antibody (1:500, Merck Millipore, Darmstadt, Germany), anti-APC antibody (CC-1, 1:500, Calbiochem Merck Millipore, Darmstadt, Germany), anti-GFAP antibody (1:1000, Sigma Aldrich, St. Louis, MO, USA), anti-Iba-1 antibody (1:500, WAKO), anti-CD31 antibody (1:500, BD, Franklin Lakes, NJ, USA), or claudin-5 (1:1000, GeneTex, Irvine, CA, USA). To assess axonal degeneration in the current study, the SMI-32 antibody reacted to non-phosphorylated neurofilaments and was used as a marker for axonal degeneration. In healthy myelinated axons, neurofilaments are not stained by SMI-32 antibody because they are heavily phosphorylated. SMI-32 immunoreactivity can provide a sensitive marker for demyelination or axonal pathologic changes [51]. In addition, several anti-MBP antibodies were used. The monoclonal antibody (SMI-94) binds all four isoforms (21.5, 18.5, 17, and 14kDa) of MBP on blots, whereas the monoclonal antibody (MCA-7D2) binds only the largest 21.5 kDa and 18.5 kDa form of MBP in rodents. Moreover, the monoclonal antibody (QD9) binds only degenerating myelin [19,20]. Immunostaining was performed following a standard fluorescein protocol. Briefly, sections were blocked with 2% normal goat serum, 5% BSA, and 0.2% tritonX-100, and then incubated with primary antibodies at 4 °C overnight. For dual staining, Alexa-488 and Alexa-594 (Molecular Probes/Invitrogen, Eugene, OR, USA)-conjugated secondary antibodies were used to visualize primary antibodies. Inflammatory foci was defined as presence of >20 inflammatory cells in the perivascular space of a given blood vessel on the HE staining [52,53]. For simple evaluation of inflammatory cells, a modified similar method by DAPI staining was adapted in the current study [12,50]. The sections were analyzed with a confocal laser microscope (FV-1000D, OLYMPUS, Tokyo, Japan) with software (Fluoview, OLYMPUS, Tokyo, Japan). Each pathology group included tissue sections from three to four animals.

### 4.4. Immunoblotting

Tissues were lysed in RIPA buffer (1% Nonidet P-40, PMSF, EDTA in PBS) [12]. After determination of the protein concentration (DC protein assay kit; BioRad, Hercules, CA, USA), 10–20 µg of protein extract was separated by 10–15% sodium dodecyl sulfate-polyacrylamide gel electrophoresis (SDS-PAGE), transferred to PVDF paper (Millipore, Darmstadt, Germany), and immunostained with the primary antibody described above or anti-GAPDH antibody (Sigma Aldrich, St. Louis, MO, USA, 1:1000). For detection, HRP-conjugated secondary antibodies (1:1000, GE Healthcare, Sunnyvale, CA, USA) were used, followed by ECL chemiluminescence development (GE Healthcare, Sunnyvale, CA, USA) with a lumino image analyzer (LAS-3000; FUJIFIRM, Tokyo, Japan).

### 4.5. Osmium-Maceration Scanning Electron Microscope (SEM) Analysis

Animals were perfused with saline followed by a mixture of 0.5% glutaraldehyde and 0.5% paraformaldehyde (PFA) in 0.1 M PB [11,12,26]. Then, the spinal cord was removed and further fixed with 0.1% OsO_4_ in 0.1 M PB. Furthermore, the spinal cord was frozen and cracked. For maceration, samples were immersed in 0.1 M OsO_4_ in 0.1 M PB. Samples were then dehydrated and dried in a critical point dryer and observed in a SEM (S-4100; Hitachi, Tokyo, Japan).

### 4.6. ES-Derived Oligodendrocyte Cell Culture

Mouse embryonic stem cell-derived oligodendrocyte progenitor cells (OPCs) were generated as previously reported [12]. OPCs were then plated onto poly-L-lysine-coated dishes (BD Biosciences, Franklin Lakes, NJ, USA) or microcoverslips and were further stimulated with 30 ng/mL triiodothyronine (T3, Sigma Aldrich, St. Louis, MO, USA) for their maturation. At 7 days, the mature OLs were exposed to tunicamycin (Tm; 1 μg/mL, Sigma-Aldrich, St. Louis, MO, USA), an ER stress inducer, for 24 h. Then the cells were subjected to either immunocytochemistry or immunoblotting.

### 4.7. Statistical Analysis

The data in this text represent the mean ± SEM, and the error bars in the figures also represent SEM. Unpaired *t*-tests were used to compare the significance of differences between two groups, and one-way ANOVA followed by Bonferroni tests were used to analyze data with more than two groups. *p <* 0.05 was considered statistically significant.

## 5. Conclusions

In conclusion, the current study clarified a part of the mechanism regarding the BBB breakdown from remission to relapse. Infiltration of peripheral inflammatory cells into the CNS occurs from areas of the BBB breakdown with reduced expression of claudin-5 or newly formed blood vessels indicated by CD31+ve angiogenesis. It is suggested that inflammatory cell infiltration from the periphery may be occurring in these areas. In addition, it is also proposed that 21 kDa MBP induced in demyelinating regions from remission to relapse is degenerated by ER-stress, resulting in the formation of abnormal myelinated structures. The regulation of the local environmental pathogenesis of ER stress-mediated degenerated MBP may be the key to the development of novel therapies for RRMS.

## Data Availability

All data produced for this manuscript are available from the lead contact (Y.B) upon reasonable request.

## Abbreviations

Abbreviations used in this paper: BBB, blood–brain barrier; CNS, central nervous system; DAPI, 4′,6-diamidino-2-phenylindole; EAE, experimental autoimmune encephalomyelitis; GFAP, glial fi-brillary acid protein; Iba-1, ionized calcium-binding adapter molecule 1, MBP, myelin basic protein; MOG, myelin oligodendrocyte glycoprotein; MS, multiple sclerosis; OLs, oligodendrocytes; OPC, oligodendrocyte precursor cell; PBS, phosphate buffered saline; PFA, paraformaldehyde.
